# Comparison of the Efficacy and Safety of Axi-Cel and Tisa-Cel Based on Meta-Analysis

**DOI:** 10.7150/jca.99427

**Published:** 2024-09-09

**Authors:** Chengcheng Liao, Lin Zeng, Shengjuan Lu, Shaocu Zheng, Baoping Guo, Qing Ke, Mingyue Wang, Jie Sun, Chao Rong, Sha He, Dani Zhong, Mei Huang, Xiaohong Tan, Hong Cen

**Affiliations:** 1 Department of Hematology/Oncology, Guangxi Medical University Cancer Hospital, Nanning, Guangxi, 530021, China.; 2 State Key Laboratory of Targeting Oncology, Guangxi Medical University, Nanning, Guangxi, 530021, China.; 3 Department of Thyroid and Breast Surgery, The First Affiliated Hospital of Guangxi University of Chinese Medicine, Guangxi, Nanning, 530022, China.; 4 Department of Pharmacy Foresea Life Insurance Guangxi Hospital, Nanning, Guangxi, 530200, China.; 5 College of Oncology, Guangxi Medical University,Nanning, Guangxi, 530021, China.

**Keywords:** Axi-cel, Tisa-cel, Efficacy, Safety, Meta-analysis, CAR-T Therapy, Chimeric Antigen Receptor T Cells (CAR-T), B-Cell Lymphoma

## Abstract

This study aimed to analyze the efficacy and safety of chimeric antigen receptor T-cell (CAR-T) therapy for B-cell lymphoma using published literature data. Literature on CAR-T therapy for B-cell lymphoma was collected by searching common databases. The literature was screened, quality assessed, and data extracted according to the inclusion and exclusion criteria. We performed a quantitative meta-analysis of the efficacy and safety of combined literature data. If the data could not be combined, descriptive analysis was performed. The meta-analysis results indicated that compared with tisagenlecleucel (tisa-cel), axicabtagene ciloleucel (axi-cel) had higher objective response rate (ORR) and complete response rate, with odds ratio (OR) of 0.63 for both sides (95% confidence interval [CI], 0.50-0.79) and statistically significant differences. Partial response rate was lower with axi-cel than with tisa-cel, with an OR of 1.02 for tisa-cel versus axi-cel (95% CI, 0.75-1.40) and no statistically significant difference. Compared with tisa-cel, axi-cel had longer progression-free survival and overall survival, with risk ratios of 0.70 (95% CI, 0.62-0.80) and 0.71 (95% CI, 0.61-0.84) for axi-cel and tisa-cel, respectively. Compared with tisa-cel, axi-cel had higher incidence rates of cytokine release syndrome (CRS) and immune effector cell-related neurotoxicity syndrome (ICANS), with ORs of 3.84 (95% CI, 2.10-7.03) and 4.4 (95% CI, 2.81-6.91), respectively. CAR T-cell therapy is an effective treatment option for relapsed/refractory B-cell lymphoma. Axi-cel has better ORR and survival advantages compared with tisa-cel; however, axi-cel has higher incidence rates of CRS and ICANS compared with tisa-cel.

## 1. Introduction

Lymphoma is a malignant tumor that originates in the blood, lymph nodes, and lymphatic tissues. Based on its histopathological characteristics, lymphoma can be divided into two main categories: Hodgkin's lymphoma and non-Hodgkin's lymphoma (NHL). The incidence rate of NHL is significantly higher than that of Hodgkin's lymphoma. NHL can be classified into B-cell, T-cell, and natural killer-cell lymphomas based on tumor cell origin, with B-cell lymphomas accounting for 70-85% of all lymphoma cases. The most common subtypes of B-cell lymphoma include diffuse large B-cell lymphoma (DLBCL), follicular lymphoma (FL), marginal zone B-cell lymphoma, and mantle cell lymphoma (MCL). Commonly used treatment options for B-cell lymphoma include chemotherapy, targeted therapy, and hematopoietic stem cell transplantation (HSCT). With the development of diagnostic techniques and new drugs, the cure rate of patients has gradually improved. However, clinical needs remain unmet. Owing to the cell toxicity and nonselective mechanism of chemotherapy drugs, several chemotherapy-related adverse reactions, including bone marrow suppression, infection, and gastrointestinal reactions, which affect the full dose and course of chemotherapy treatment to a certain extent, are observed. Anti-CD20 monoclonal antibodies have improved the prognosis of CD20+ B-cell lymphoma and demonstrated the feasibility and effectiveness of immunotherapy in B-cell lymphoma 1, 2. However, some patients may still develop resistance 3-7. HSCT can effectively prolong the progression-free survival (PFS) and overall survival (OS) of patients with chemotherapy-sensitive lymphoma. However, owing to patient age, physical condition limitations, or chemotherapy resistance, some patients with relapsed/refractory (R/R) disease cannot undergo HSCT or benefit from it. Therefore, better treatment options for this condition need to be explored. CAR-T immunotherapy is currently a popular research area, and its emergence has provided new opportunities for the treatment of B-cell lymphoma 6-8.

CAR-T therapy mainly uses genetic engineering technology to reorganize and recognize the single-chain fragment variable of tumor-associated antigens and the intracellular signaling domain “immune receptor tyrosine activation motif” *in vitro* and then transfect the recombinant plasmid into T cells of the recipient through gene transfer technology. After CAR-T cells recognize and bind to tumor cell surface antigens, signal transduction pathways are activated in T cells and tumor cell apoptosis is triggered through a series of reactions, thus exerting antitumor effects. In 2013, the *New England Journal of Medicine* reported that two patients with R/R acute lymphoblastic leukemia (ALL) achieved complete remission after receiving anti-CD19 CAR-modified T-cell adoptive transfer therapy. CAR-T therapy provides a new treatment option for prolonging survival and improving the prognosis of patients with R/R disease, with significant clinical application prospects 9. Subsequently, an increasing number of clinical trials on CAR-T therapy have been conducted, and CAR-T cells targeting CD33, CD20, CD30, CD123, ERRB2, and other targets have been developed and applied. As of March 2023, eight CAR-T products have been approved for clinical application by the US Food and Drug Administration and National Medical Products Administration. As of February 2023, there are 2517 CAR-T-related clinical studies registered on clinical trials.gov 10, 11.

However, certain factors have hindered the development of CAR-T therapy. First, several factors may lead to the failure of CAR-T therapy, including its toxic side effects, such as CRS and ICANS. CRS is the most common adverse reaction in cellular immunotherapy, triggered by tumor cell-associated antigens and activated T cells following CAR-T binding 10, 11, and is characterized by high fever, tachycardia, hypotension, hypoxia, cardiac depression, and other organ dysfunctions 12. ICANS is a neurological toxicity caused by immune effector cells, such as CAR-T cells, which manifests as a series of clinical symptoms in the nervous and mental systems, including changes in mental status, aphasia, varying degrees of consciousness disorders, hemiplegia, and epilepsy 13, 14. Although current experience and techniques for managing CAR-T-related adverse reactions are relatively mature, early suppression of CRS or ICANS may limit the activity and proliferation capacity of CAR-T cells and thus cause loss of efficacy. Additionally, severe adverse reactions can endanger patients' lives and cause sequelae and long-term toxicity 15, 16.

The results of different CAR-T products in terms of therapeutic efficacy and safety are different, and there remains a lack of studies on the comparison of the efficacy and safety of different CAR-T products or combinations. Moreover, appropriate selection among different CAR-T therapies remains unclear. Therefore, to evaluate the efficacy and safety of commonly used CAR-T therapies, this study aimed to conduct a systematic review of the efficacy and safety of CAR-T therapy for B-cell lymphoma (MCL, DLBCL, FL, etc.) using published literature data and conduct a meta-analysis of high-quality controlled studies.

## 2. Materials and methods

### 2.1 Literature retrieval strategy

We searched using the Population, Intervention, Comparison, Outcomes, and Study design principles: Population: B-cell lymphoma (MCL, DLBCL, FL, etc.); Intervention: CAR-T therapy; Outcome indicators: primary outcome indicators (clinical efficacy, ORR, CR, PR, OS, PFS) and secondary outcome indicators (adverse reactions: CRS and ICANS); and Study design: Efficacy and safety studies.

### 2.2 Development of search formulas

The Scopus, Web of Science (WOS), China National Knowledge Infrastructure (CNKI), Chongqing VIP Information(cqVIP), Wanfang Medicine, Chinese biomedical literature database, and PubMed databases were searched from the establishment of the database to March 31, 2023, without language restrictions. The search method of subject headings and free words was used.

### 2.3 Inclusion and exclusion criteria

The inclusion criteria were as follows: (1) studies assessing patients diagnosed with B-cell lymphoma (MCL, DLBCL, FL, etc.); (2) studies assessing patients who received CAR-T treatment (monotherapy or combination therapy); (3) studies with controlled study as the study method; and (4) studies with ORR, CR, PR, OS, and PFS as the primary outcome indicators and CRS and ICANS as the secondary outcome indicators. The exclusion criteria were as follows: (1) studies with repeated publication or without full text and valid data; (2) noncontrolled studies, case reports, reviews, meta-analyses, and other secondary studies; and (3) studies with total sample sizes of < 10 cases.

### 2.4 Literature screening

The retrieved literature was imported into EndNote X7 software for deduplication, and the deduplicated literature was screened by two lymphahematologists according to the inclusion and exclusion criteria and cross-checked. If any controversial issues arose, a third professional was consulted to resolve them.

### 2.5 Quality evaluation of the literature

The cohort study was evaluated using the Newcastle-Ottawa Scale. According to the scores, the literature can be divided into three grades: low, medium, and high. The results of the quality assessment of the above methods were low, and the studies were not included in this analysis. In case of disagreement, a third investigator was consulted, and a consensus was reached.

### 2.6 Data extraction from the literature

The following information was recorded in the included studies: author, year, study type, sample size, treatment protocol, and outcome indicators.

### 2.7 Data analysis

If two or more studies reported the same outcome indicator, a meta-analysis was performed using the “meta (version 6.5-0)”, “gemtc (version 1.0-2)” and “rjags (version 4-15)” software packages of R software 3.6.1. Binary variables are expressed as odds ratios (ORs) and their 95% confidence intervals (CIs). Survival data are expressed as hazard ratios (HRs) and their 95% CIs. If the original literature did not provide the HRs, we used the Engauge-Digitizer (Version 8.3) data extraction software to extract data from survival curves and Tierney's summary method to calculate the HRs 17, 18. When a quantitative meta-analysis was not feasible because only a single article reported the same outcome or heterogeneity between study populations, we performed a descriptive analysis of the outcome indicator only.

### 2.8 Heterogeneity analysis

We used the chi-squared test to calculate the I^2^ and P values. If the Q test results showed I^2^ > 50% or P < 0.05, indicating significant heterogeneity among studies, we used a random-effects model for analysis, and subgroup or sensitivity analysis was performed to detect bias and further discuss the heterogeneity. Conversely, if no heterogeneity was found among the studies, a fixed-effects model was used for the analysis.

### 2.9 Bias test

When the number of studies included in the meta-analysis was > 10, the bias test was performed, and funnel plots were used to analyze the bias of the included studies using the R software 3.6.1 “meta” package.

## 3. Results

### 3.1 Literature search results

**Figure 1:**
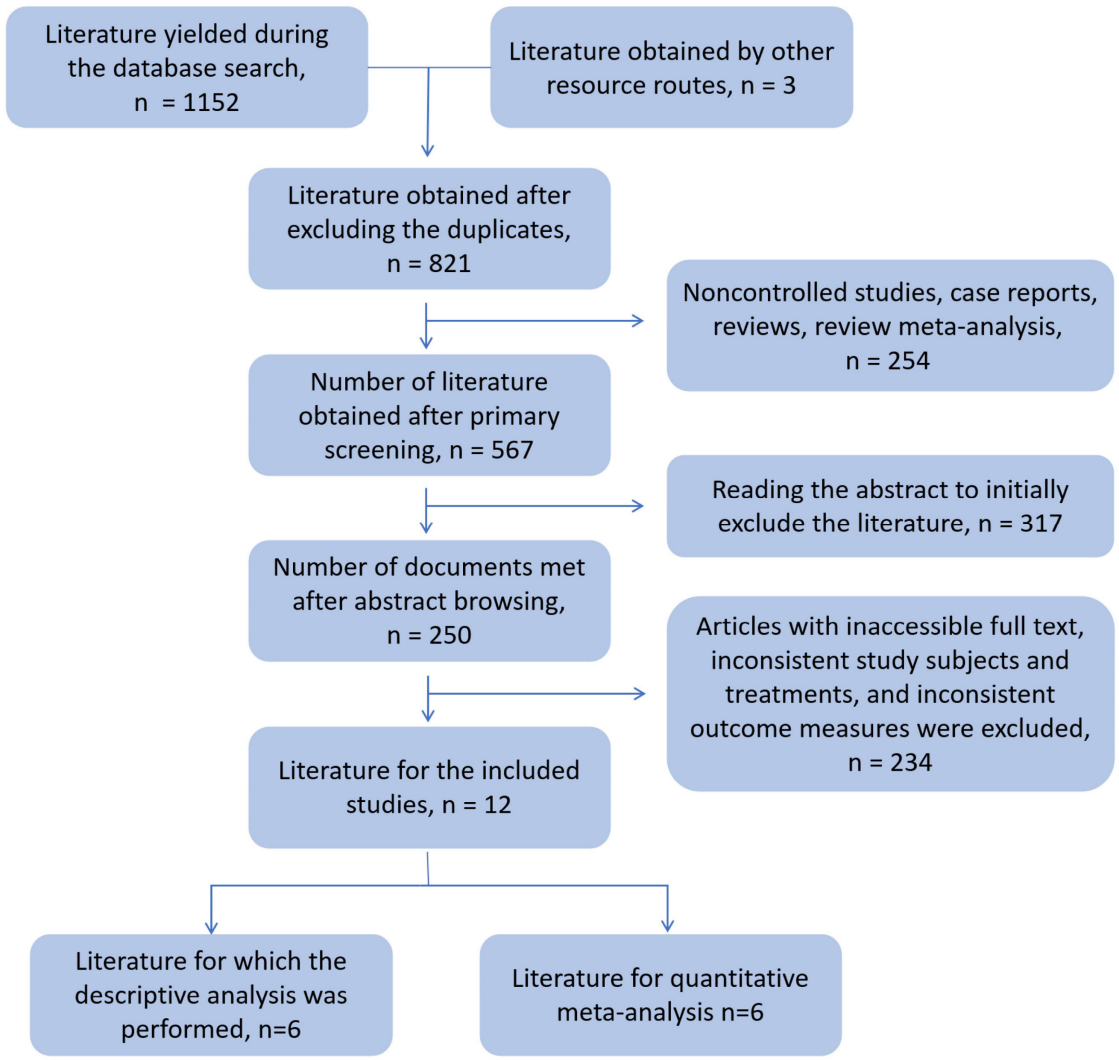
Document screening flowchart

The literature selection process is shown in Figure 1; 16 studies were included in the final analysis. Among them, there were six articles on tisa-cel and axi-cel treatment control, which could be quantitatively meta-analyzed. The results of the remaining 10 controlled studies were analyzed using descriptive analysis.

### 3.2 Basic information on the literature included in the quantitative meta-analysis

The basic information of the six articles is shown in Table 1.

### 3.3 Quality evaluation

In summary, the six articles of tisa-cel and axi-cel treatment control were all cohort studies assessed using the Newcastle-Ottawa Scale, and the six articles were all medium- and high-quality literature. The results of the quality evaluation are presented in Table 2.

### 3.4 Statistical results

#### 3.4.1 Quantitative meta-analysis results

##### 3.4.1.1 Objective response rate of axi-cel and tisa-cel treatments

**Figure 2:**
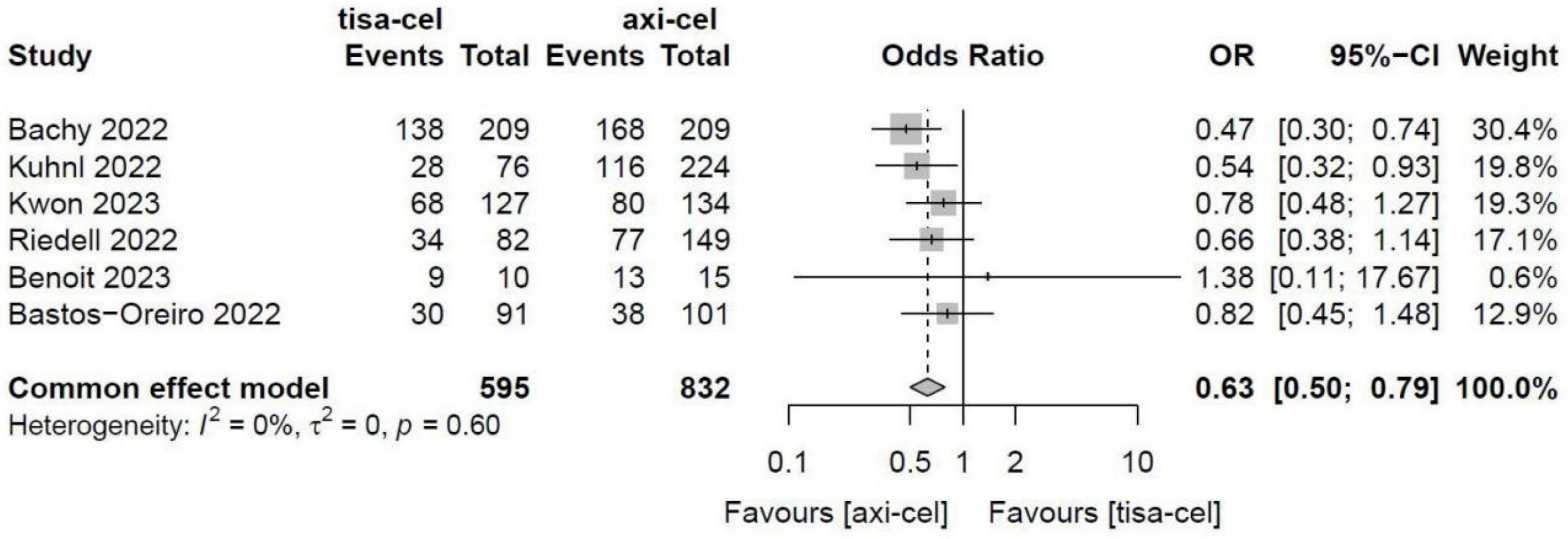
Forest plots of objective response rate in a fixed-effects model

The ORRs of tisa-cel and axi-cel were presented in all six studies (I^2^ = 0.0%, P = 0.60), and there was no heterogeneity among the studies. The overall ORRs were 51.5% for tisa-cel and 59.1% for axi-cel. With an OR of 0.63 (95% CI, 0.50-0.79) based on a fixed-effect model, axi-cel was associated with a statistically significant ORR compared with tisa-cel (Figure 2).

##### 3.4.1.2 Complete response rates (CRRs) of tisa-cel and axi-cel treatments

**Figure 3:**
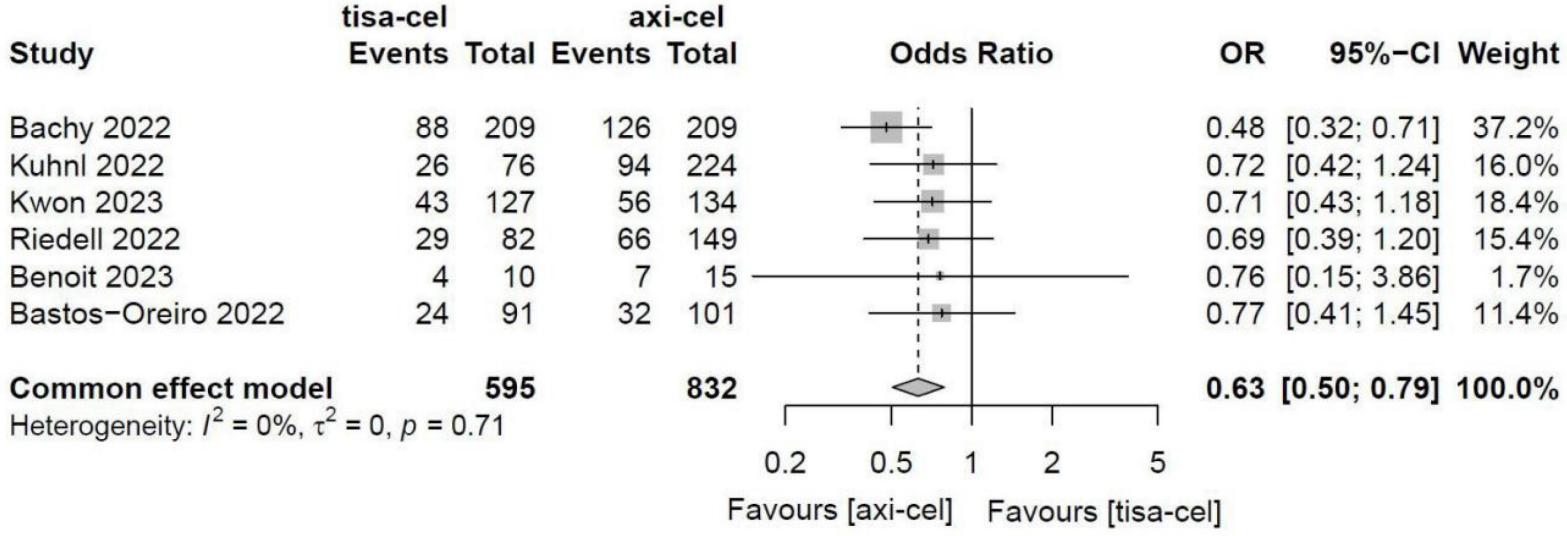
Forest plots of complete remission rate in a fixed-effects model

The CRRs of tisa-cel and axi-cel were presented in all six studies (I^2^ = 0.0%, P = 0.71), and there was no heterogeneity among the studies. The overall CRR for tisa-cel and axi-cel were 36.0% and 45.8%, respectively. The OR was 0.63 (95% CI, 0.50-0.79) according to a fixed-effect model. Axi-cel had higher CRR compared with tisa-cel, and the difference was statistically significant (Figure 3).

##### 3.4.1.3 Partial response rates (PRRs) of tisa-cel and axi-cel treatments

**Figure 4:**
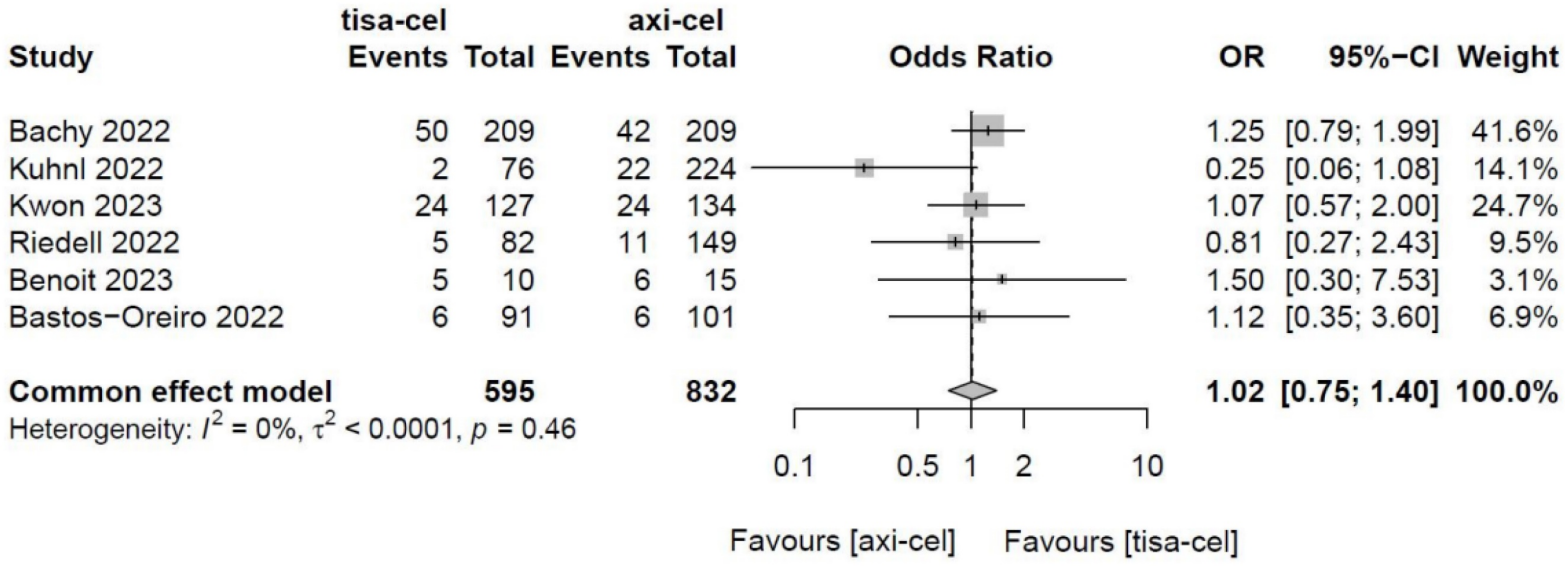
Forest plots of partial remission rate in a fixed-effects model

The PRRs of tisa-cel and axi-cel were presented in all six studies (I^2^ = 0.0%, P = 0.46), with no heterogeneity among the studies. The overall PRRs were 15.5% for tisa-cel and 13.3% for axi-cel.^22^ According to a fixed-effect model,^23^ the OR was 1.02 (95% CI, 0.75-1.40), and axi-cel had lower PRR compared with tisa-cel (Figure 4).

##### 3.4.1.4 PFS of tisa-cel and axi-cel

**Figure 5:**
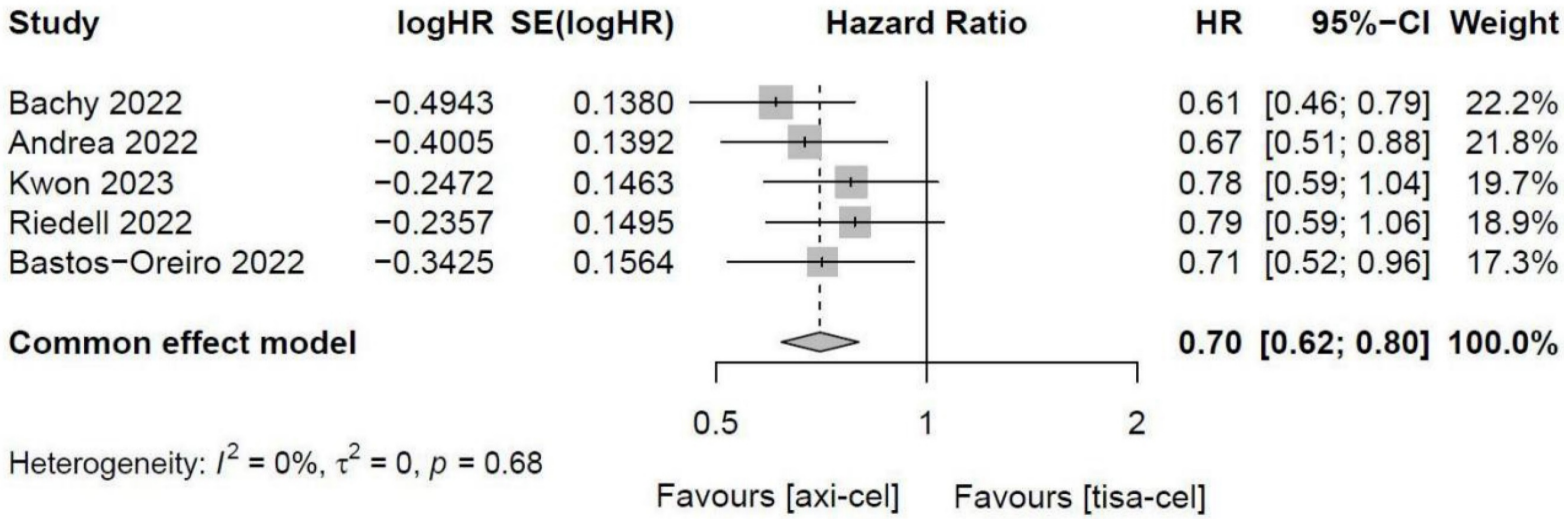
Forest plots of progression-free-survival in a fixed-effects model

Five of the included studies 19-23, 27 presented the PFS rates of tisa-cel and axi-cel (I^2^ = 0.0%, P = 0.68), with no heterogeneity among the studies. In five studies, the 1-year PFS rates ranged from 41.0% to 46.6% for axi-cel and from 27.4% to 33.2% for tisa-cel. With an HR of 0.70 (95% CI, 0.62-0.80) according to a fixed-effect model, axi-cel had a longer PFS compared with tisa-cel, and the difference was statistically significant (Figure 5).

##### 3.4.1.5 Total OS of tisa-cel and axi-cel treatments

**Figure 6:**
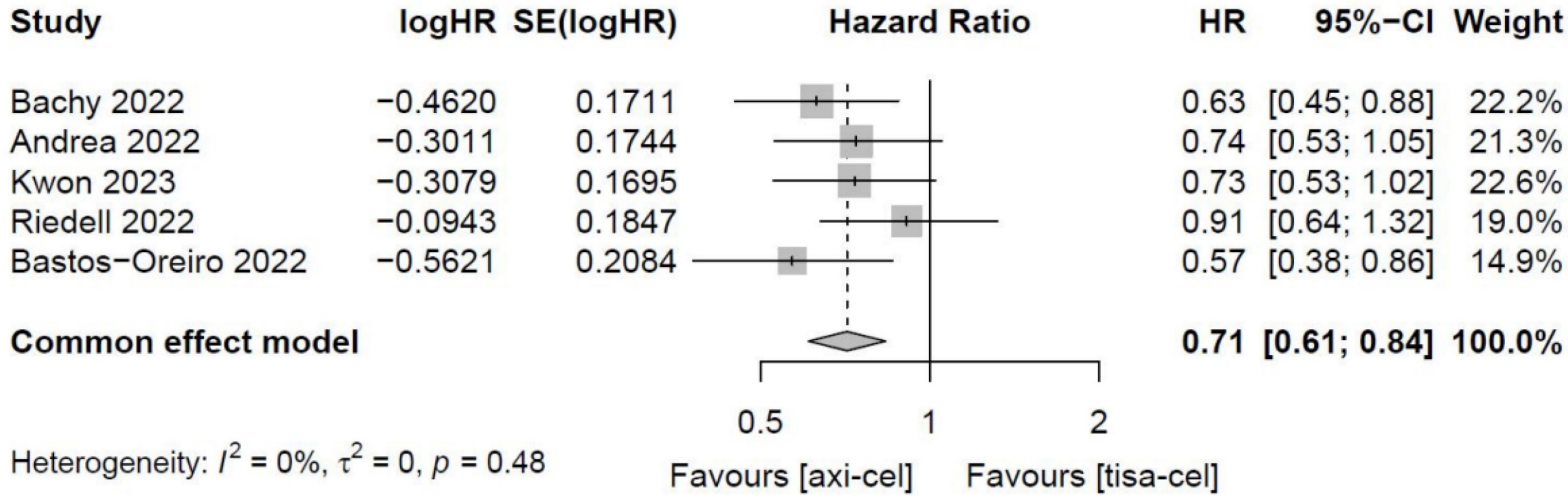
Forest plots of overall survival in a fixed-effects model

Five of the included studies 19-23 presented the OS rates of tisa-cel and axi-cel (I^2^ = 0.0%, P = 0.48), and there was no heterogeneity among the studies. In five studies, the 1-year OS rates ranged from 51.0% to 63.5% for axi-cel and from 43.8% to 59.0% for tisa-cel. According to the fixed-effect model, the HR was 0.71 (95% CI, 0.61-0.84), and axi-cel had longer OS compared with tisa-cel, and the difference was statistically significant (Figure 6).

##### 3.4.1.6 Main toxicity of CAR-T therapy

3.4.1.6.1 Cytokine release syndrome

**Figure 7:**
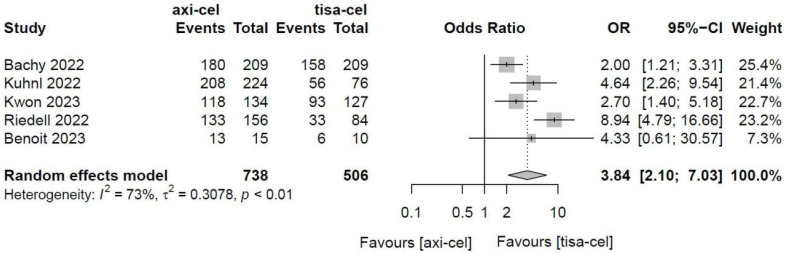
Forest plots of the rates of cytokine release syndrome in a random-effects model

**Figure 8:**
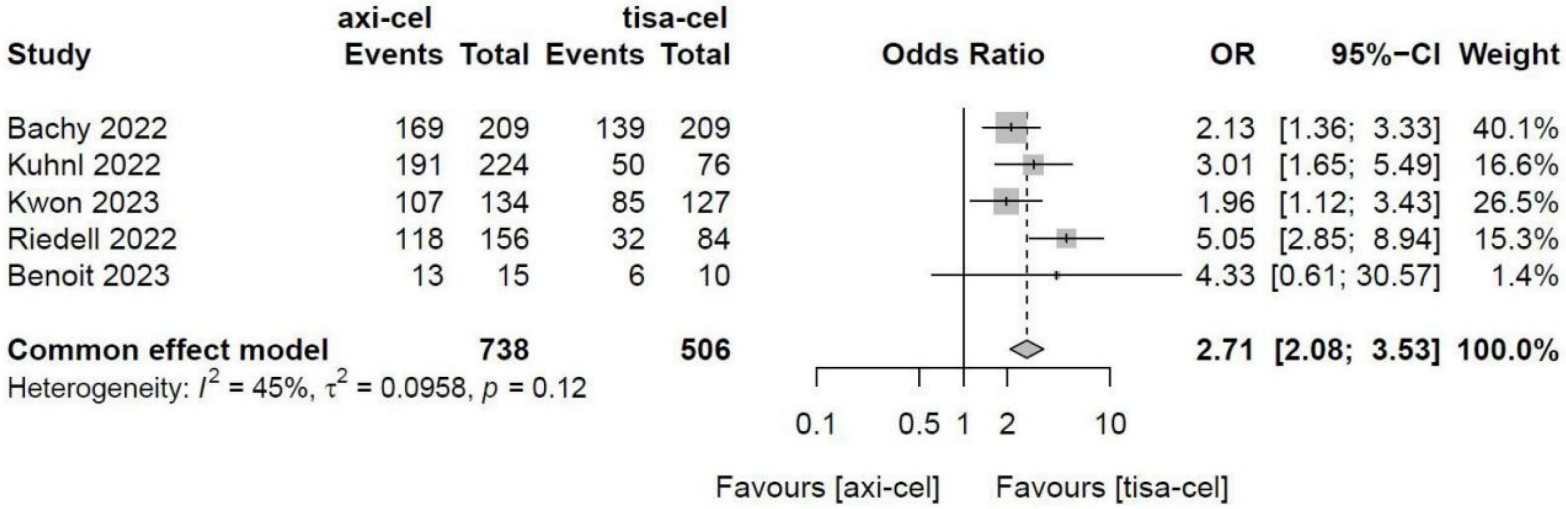
Forest plots of the rates of grade 1-2 cytokine release syndrome in a fixed-effects model

**Figure 9:**
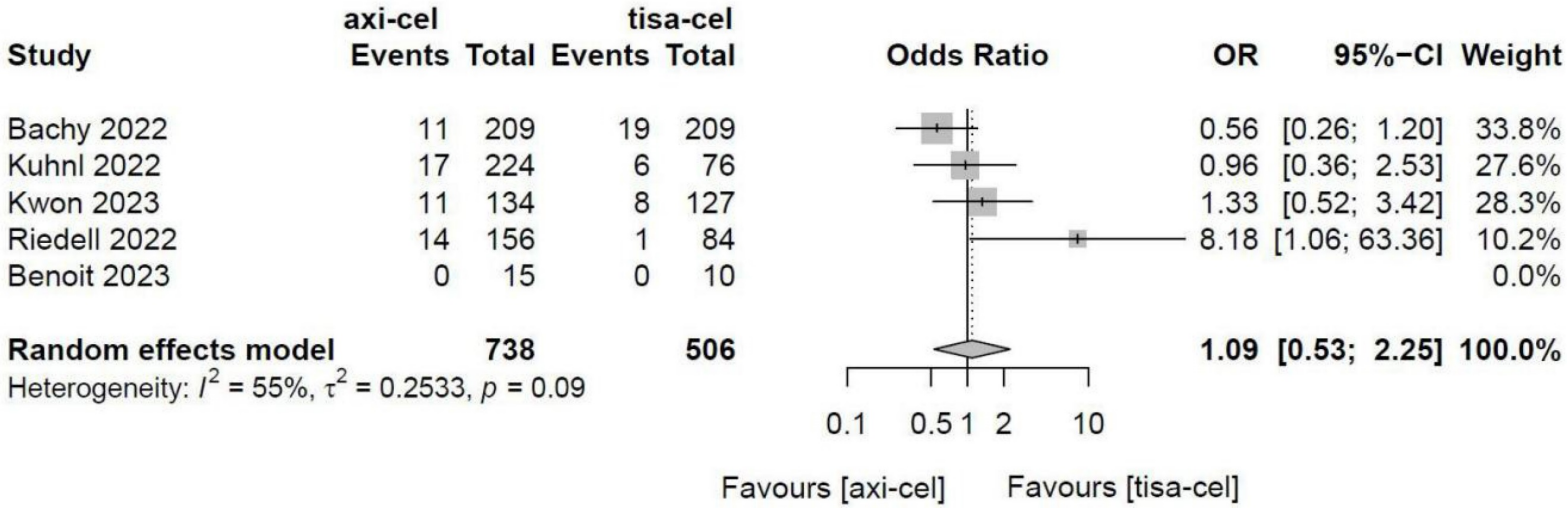
Forest plots of the rates of grade ≥ 3 cytokine release syndrome in a random-effects model

Five of the included studies 19-23 presented the incidence rates of CRS (Table S1 showed the toxicity grading method 28) treated with tisa-cel and axi-cel (I^2^ = 73%, P < 0.01); there was heterogeneity in the literature. The overall incidence rates of CRS were 88.3% and 68.4% for axi-cel and tisa-cel, respectively. With an OR of 3.84 (95% CI, 2.10-7.03) according to a random-effects model, axi-cel was associated with a statistically significantly higher CRS compared with tisa-cel (Figure 7). To determine the source of heterogeneity, subgroup analysis of CRS severity was performed, and the incidence rates of grade 1-2 CRS and ≥ 3 CRS were determined. Regarding the incidence rates of grade 1-2 CRS, the calculated results suggested an I^2^ value of 45% and a P value of 0.12, and there was no heterogeneity among the studies. The overall incidence rates of grade 1-2 CRS for axi-cel and tisa-cel were 81.1% and 61.7%, respectively. With an OR of 2.71 (95% CI, 2.08-3.53) according to a fixed-effect model, axi-cel had a statistically significantly higher incidence rate of grade 1-2 CRS compared with tisa-cel (Figure 8). In grade ≥ 3 CRS, the results suggested an I^2^ value of 55% and a P value of 0.09, indicating a slight heterogeneity among the literature. The overall incidence rates of grade ≥ 3 CRS were 7.2% for axi-cel and 6.7% for tisa-cel. With an OR of 1.09 (95% CI, 0.53-2.25) according to the random-effects model, axi-cel had a higher incidence rate of grade ≥ 3 CRS compared with tisa-cel, but the difference was not statistically significant (Figure 9). Other common adverse toxicities showed in Table 3.

3.4.1.6.2 Immune effector cell-associated neurotoxicity syndrome

**Figure 10:**
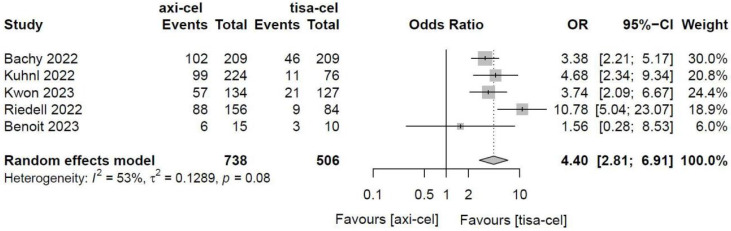
Forest plots of the rates of immune effector cell-related neurotoxicity syndrome in a random-effects model

**Figure 11:**
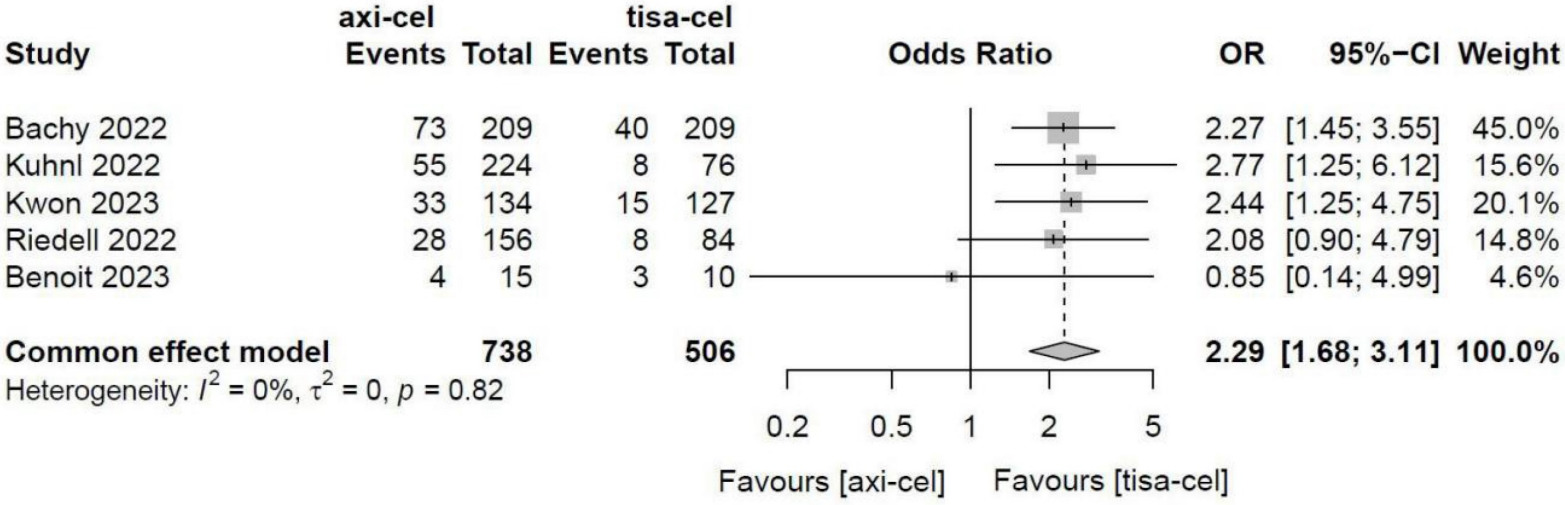
Forest plots of the rates of grade 1-2 immune effector cell-related neurotoxicity syndrome in a fixed-effects model

**Figure 12:**
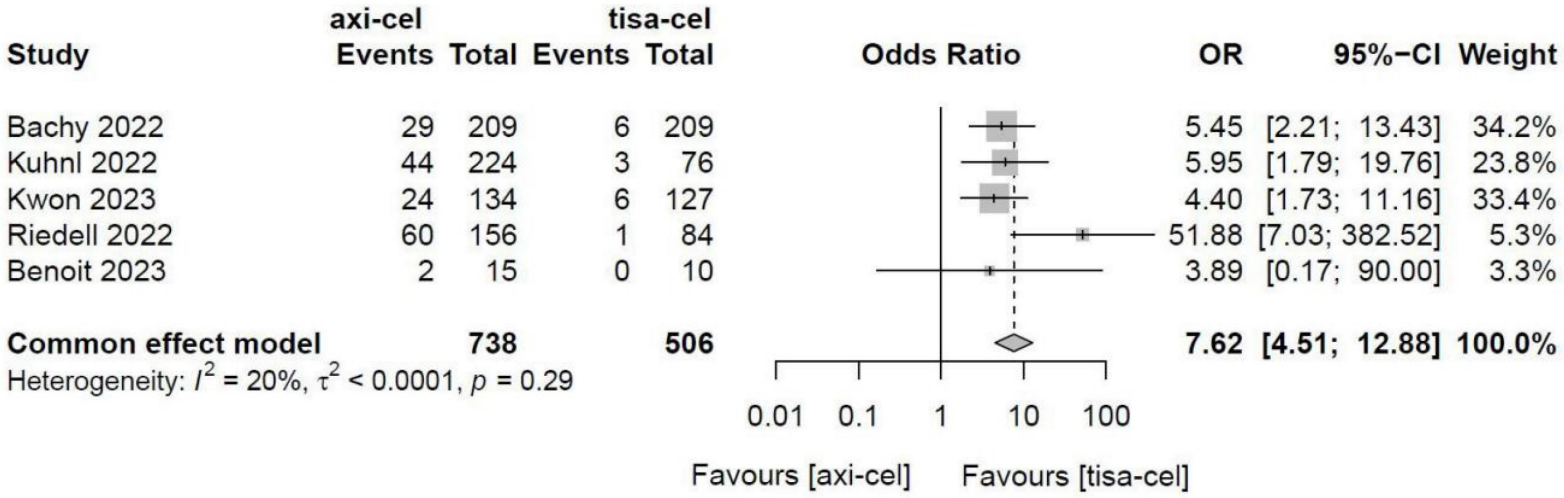
Forest plots of the rates of grade ≥ 3 immune effector cell-related neurotoxicity syndrome in a fixed-effects model

Five of the included studies 19-23 presented the incidence rates of ICANS (Table S1 showed the toxicity grading method) treated with tisa-cel and axi-cel, and meta-analyses suggested an I^2^ value of 53% and a P value of 0.08, with mild heterogeneity among the literature. The total incidence rates of ICANS were 47.7% and 17.8% for axi-cel and tisa-cel, respectively. With an OR of 4.4 (95% CI, 2.81-6.91) according to the random-effects model, axi-cel had a statistically significantly higher ICANS rate compared with tisa-cel (Figure 10). To determine the source of heterogeneity, subgroup analyses were performed to analyze the severity of ICANS, and the incidence rates of grade 1-2 ICANS and ≥ 3 ICANS were identified. Regarding the incidence rate of grade 1-2 ICANS, the calculated results suggested an I^2^ value of 0.0% and a P value of 0.82, and there was no heterogeneity among the studies. The incidence rates of grade 1-2 ICANS were 26.2% for axi-cel and 14.6% for tisa-cel. With an OR of 2.29 (95% CI, 1.68-3.11) according to a fixed-effect model, axi-cel had a statistically significantly higher incidence rate of grade 1-2 ICANS compared with tisa-cel (Figure 11). In grade ≥ 3 ICANS, the results suggested an I^2^ value of 20% and a P value of 0.82, and there was no heterogeneity among the studies. The incidence rates of grade ≥ 3 ICANS were 21.5% for axi-cel and 3.2% for tisa-cel. The OR was 7.62 (95% CI, 4.51-12.88) according to the fixed-effect model, with a statistically significant difference in the incidence rate of grade ≥ 3 ICANS between axi-cel and tisa-cel (Figure 12).

#### 3.4.2 Publication bias

As there were < 10 studies in the quantitatively pooled meta-analysis for each index, we concluded that there was no publication bias between the studies, and no publication bias assessment was required.3.4.2 Quantitative meta-analysis and MeSH meta-analysis results (CRR)Three RCTs were used for network meta-analysis (axi-cel and liso-cel, tisa-cel and liso-cel, SOC and liso-cel), with ORs of 0.89 (95% CI, 0.26-3.00) for the comparison between axis-cel and liso-cel, 0.53 (95% CI, 0.15-1.80) for the comparison between tisa-cel and liso-cel, and 0.33 (95% CI, 0.12-0.92) for the comparison between SOC and liso-cel. Therefore, based on the results, the CRR of the axis-cel was higher, followed by the tisa-cel, and the SOC was the worst, but the difference was not statistically significant, as shown in the supplementary figure.

#### 3.4.3 Qualitative descriptive analysis results

The basic literature information of the 10 controlled studies is shown in Table S2. Salles et al. 29 showed that tisa-cel therapy was more effective than the conventional third-line therapy for R/R FL. Sermer et al. 30 showed that in R/R DLBCL, the efficacy of CAR-T therapy was superior to that of alternative therapy (the most commonly used third-line regimen was a platinum-based regimen [29%], investigational regimen [26%], etoposide regimen [8.9%], and anthracycline-based regimen [8.9%]).Avivi et al. 31 showed that the CAR-T arm was more effective than the polatuzumab vedotin (Pola)-based regimen for treating R/R DLBCL. Locke et al. 25 showed that axi-cel treatment was more effective than platinum-based salvage chemotherapy combined with SOC for autologous HSCT in R/R B-cell lymphoma. Kamdar et al. 26 showed that liso-cel treatment was more effective than SOC treatment for R/R B-cell lymphoma. Bishop et al. 27 showed that tisa-cel was more effective than SOC in treating R/R NHL. Yan et al. 32 showed that patients with R/R B-ALL treated with CAR-T cells had a slightly higher efficacy than CD3 CD19 bispecific antibodies and was significantly higher than that in the traditional chemotherapy group.

## 4. Discussion

The current treatment options for B-cell lymphoma include chemotherapy, targeted therapy, and HSCT. However, there are several chemotherapy-related adverse reactions due to the cytotoxic and nonselective mechanism of action of chemotherapy drugs, which affect the full dose and course of chemotherapy treatment to a certain extent.

The use of anti-CD20 monoclonal antibodies has improved the prognosis of CD20+ B-cell lymphoma; however, resistance persists in some patients. Owing to age, physical condition limitations, and chemotherapy resistance, some patients cannot undergo or benefit from HSCT. Therefore, better treatment options for this condition need to be explored. The emergence of CAR-T technology has provided new opportunities for the treatment of lymphatic and hematopoietic tumors. As of 2022, six different CAR-T products have been approved for clinical application by the US Food and Drug Administration and National Medical Products Administration. The therapeutic efficacy and safety of different CAR-T products differ, and there remains a lack of studies on the comparison of the efficacy and safety of different CAR-T products or combinations. Moreover, appropriate selection among different CAR-T therapies remains unclear. Therefore, we aimed to perform a meta-analysis using published literature data on the efficacy and safety of CAR T-cell therapy for B-cell lymphoma. Through meta-analysis, it can be observed that among the different CAR-T designs, efficacy is also different. The ORR and CRR of axi-cel were higher than those of tisa-cel. The overall ORRs were 59.1% for axi-cel and 51.5% for tisa-cel, whereas the overall CRRs were 45.8% for axi-cel and 36.0% for tisa-cel. Notably, the PRR of axi-cel was lower than that of tisa-cel; however, considering that the ORR and CRR of axi-cel were both higher than those of tisa-cel, we hypothesized that this was due to the higher CRR rate of the axi-cel treatment. We used three RCTs to perform a network meta-analysis on liso-cel, axi-cel, and tisa-cel to indirectly compare the difference in efficacy between liso-cel and the other two CAR-T products. However, owing to the loss of statistical performance caused by the sample size, number of clinical studies, and indirect comparison, no statistically significant results were obtained. Further clinical studies are required to answer these questions.

Overall, we believe that axi-cel is more effective than tisa-cel. Although CAR-T treatment has good efficacy in R/R B-cell lymphoma, its adverse reactions also need to be monitored to better benefit from it. The most common adverse reactions associated with CAR-T treatment are CRS and ICANS. Therefore, we performed a meta-analysis on the incidence rates of CRS and ICANS for axi-cel and tisa-cel. In our study, the incidence rates of CRS and ICANS in axi-cel were higher than those in tisa-cel. To exclude heterogeneity, we further performed a subgroup analysis according to severity grading, and the results indicated that in the same level group, axi-cel still showed a higher incidence rate than tisa-cel. Therefore, although the efficacy of axi-cel is better than that of tisa-cel, the incidence rates of CRS and ICANS are higher in axi-cel than in tisa-cel. Both these factors must be considered when making individualized assessments and treatment choices for patients. Relevant literature indicates that the prophylactic use of steroids, anakinra, and tocilizumab significantly reduces the incidence rate of severe CRS or ICANS. For example, a recent prospective evaluation study on the early use of dexamethasone in axi-cel therapy showed that the rate of grade ≥ 3 ICANS was only 17% 33. Toxicity may cause greater concern in older patients and offset higher efficacy. Bachy et al. also performed planned subgroup analyses on patients aged < 70 and > 70 years. Axi-cel therapy showed higher efficacy in different age groups for PFS and OS, and the PFS was significantly prolonged 19. Therefore, they believe that, even if the toxicity of axi-cel is high, longer PFS and OS can still be achieved.

Both axi-cel and tisa-cel are anti-CD19 CAR-T therapies with similar CAR designs and extracellular binding domains. However, they differ in the following aspects: (1) In terms of the design of the CAR-T molecule, the co-stimulatory domain of axi-cel is CD28, whereas that of tisa-cel is 4-1BB; (2) regarding CAR-T transduction, axi-cel is transduced by retrovirus, whereas tisa-cel is transduced by lentivirus. Regarding starting materials, axi-cel is a fresh leukocyte separation product, whereas tisa-cel is a frozen leukocyte separation product. The difference in CAR design, especially the co-stimulatory domain of CAR between tisa-cel and axi-cel, affects cell kinetics and cell fate immediately after infusion. The CD28 co-stimulatory domain induces faster expansion and differentiation; therefore, axi-cel has advantages in terms of efficacy. However, the 4-1BB co-stimulatory domain can lead to a lower expansion peak and longer persistence, allowing tisa-cel to reduce the incidence of acute toxicity from autologous CAR-T cells. This may explain why axi-cel had better efficacy in this study than tisa-cel, but the incidence rates of CRS and ICANS were lower in tisa-cel than in axi-cel.

Single CAR-T therapy has shown great advantages over other third-line treatments, as shown in previous studies. Sermer et al. showed that in R/R B-cell lymphoma, CAR-T therapy was more effective than other regimens (based on platinum, etoposide, anthracycline) 30. Avivi et al. suggested that CAR T-cell therapy was superior to Pola-based treatment 31. Su et al. found that in R/R B-ALL, the efficacy of CAR-T therapy was slightly higher than that of the CD3 CD19 bispecific antibody (blinatumomab) and significantly higher than that of traditional chemotherapy 32. CAR-T cells show good efficacy in the treatment of other small-molecule targeted drugs, alone or in combination.

In the real world, we should balance pharmacoeconomics, the patient's performance status, tumor burden, the timing of CART preparation, and the patient's tolerance of adverse effects to develop an appropriate treatment strategy. In some studies, such as Peter A. Riedell's study 23, we found that regardless of the cause, whether it is death from recent toxicity (Death within 28 days of infusion) or death from long-term toxicity (Death beyond 28 days of infusion), Axi-cel has a higher mortality rate due to Neurotoxicity, infection, and heart than Tisa-cel. This may be related to higher rates of CRS, ICANS, and cytopenias in Axi-cel. Several CRS prediction models 34 and data from our pooled analysis help us distinguish patients at high risk for toxic reactions. By choosing products with relatively small toxic effects such as Tisa-cel, patients can have a better experience during the treatment process. In addition, we also conducted subgroup and multiple regression analysis on each included study (see Table S3) and found that high ECOG score, lactate dehydrogenase greater than normal value, primary refractory, etc. are risk factors with higher occurrence rate. These seem to prompt us how to more efficiently screen the advantageous groups for CART treatment. It is worth mentioning that in the 2 included studies 19, 22, patients who received an autologous transplant before CART improved PFS, but not OS. Statistical significance. It tells us that some high-risk factors can be improved by other therapies (such as autologous transplantation, combination therapy with new drugs, etc.), which may improve the effectiveness of CART treatment. Furthermore, ASCT tandem CART has the opportunity to become a good way to improve patients with refractory lymphoma. These are issues worthy of our continued thinking and practice.

Another interesting issue is that the bispecific antibodies glofitamab and epcoritamab and mosunetuzumab are now approved for use in DLBCL 35. It gives new options to certain patients who cannot wait for CAR-T product manufacturing, or whose socioeconomic status is a barrier to commercial CAR-T, or who may lack enough circulating T cells for adequate blood separation. BsAb can achieve rapid response and remission, with a median of 20 months 36, but there is no convincing evidence of cure so far. This may be related to the fact that the target of bispecific antibodies is CD20 and the target of CART is CD19. The first-line treatment with rituximab (anti-CD20) weakens or even eliminates the expression of CD20 on the tumor surface, while the CD19 antigen is retained. Fortunately, the use of bispecific antibody treatment after CART failure still has a good therapeutic effect. The side effects are similar to those of CAR-T, but the frequency and severity are lower 37. In current clinical practice, CART is still the first choice for bispecific antibodies for relapsed and refractory B-cell lymphoma.

## 5. Innovation and limitations

Innovation: This study constructed a network meta model of Axi-cel, Liso-cel, Tisa-cel and standard of care, which is shown in the supplementary file. If more studies are updated, such as dual-target CART compared with single-target CART, and Axi-cel compared with autologous transplantation, they can be included in the model for indirect comparison. In addition, compared with previously published papers, our study conducted a summary analysis of various risk factors that are detrimental to survival and obtained several relatively stable risk factors, which provides support for the screening of patients with advantages in CART treatment and the construction of a prognostic index model. Limitations: In meta-analysis, some clinical studies and real-world controlled studies are combined for analysis, which may introduce bias in the results caused by imbalances in baseline and subsequent treatment methods. For example, one study included a small number of patients undergo CD19-based prior treatments based on mAbs (usually <5%) and the proportion and type of bridging regimens used were different. It is worth mentioning that we examined the lymphoma subtypes of these studies, which basically included DLBCL, Transformed FL, HGBL, PMBCL, and transformed indolent lymphoma, of which DLBCL accounted for about 64%-84%. Riedell, Kuhnl 20, Bachy 19, Bastos-Oreiro 22, and Kwon 21 were retrospective studies, and there was no statistical difference between their ARM A and ARM B in the classification of lymphoma subtypes (P>0.05). Locke 25, Kamdar 26, and Bishop 27 were prospective randomized controlled clinical trials. Due to the large differences in the number of various lymphoma subtypes, it is impossible to obtain the original data corresponding to each lymphoma subtype studied, so further subgroup analysis cannot be performed for specific lymphoma subtypes. Although this retrospective analysis has some limitations, such as the possible practice patterns and patient selection bias of specific centers, our data show that in the real world, patients treated with commercial axi-cel and tisa-cel show consistent practice patterns and results. The majority of patients achieved durable complete remissions after these treatments, even with high-risk disease, underscoring the positive impact of axi-cel and tisa-cel in treating relapsed/refractory B-cell non-Hodgkin lymphoma (R/R B-NHL). Finally, there are differences in the studies we included. In some studies, Previous treatment lines are factors that affect PFS, but do not affect OS. Now, it turns out that more and more studies move CART treatment lines forward, which may improve patients' quality of life. Survival is helpful, but the inclusion criteria in each independent study (the same study) are consistent, and the number of previous treatment lines in each treatment arm is basically similar, so there will be a small impact, but it will not affect the conclusion.

In conclusion, CAR-T therapy is an effective treatment option for patients with R/R B-cell lymphoma. In the CAR-T treatment regimen, axi-cel has better ORR and OS than tisa-cel; however, axi-cel treatment leads to higher incidence rates of CRS and ICANS than tisa-cel treatment. Treatment decisions should be made on an individualized basis after weighing the advantages and disadvantages.

## Supplementary Material

Supplementary figure.

Supplementary table 1.

Supplementary table 2.

Supplementary table 3.

## Figures and Tables

**Table 1:** The basic characteristics of the literature

Author	Year	Population	Treatment regimen		Previous	Median age(years)		Bridge therapy(n)		Research category	Outcome measures
			Arm A	Arm B	treatment lines	Arm A	Arm B	Arm A	Arm B		
Bachy 19	2022	R/R DLBCL	Tisa-cel	Axi-cel	Median previous	85	86	180	183	Cohort studies	OR/CR/PR/PFS/OS/CRS/ICANS
			n=209	n=209	lines:2						
Kuhnl 20	2022	R/R LBCL	Tisa-cel	Axi-cel	37.3% pts: ≥3	63	57	62	198	Cohort studies	OR/CR/PR/PFS/OS/CRS/ICANS
			n=76	n=224							
Kwon 21	2023	R/R DLBCL	Tisa-cel	Axi-cel	Median previous	62	59	106	104	Cohort studies	OR/CR/PR/PFS/OS/CRS/ICANS
			n=127	n=134	lines:2						
Bastos-Oreiro 22	2022	R/R LBCL	Axi-cel	Tisa-cel	Median previous	54	56	54pts in 2 Arms		Cohort studies	OR/CR/PR/PFS/OS
			n=101	n=91	lines:2						
Riedell 23	2022	R/R LBCL	Tisa-cel	Axi-cel	Tisa-cel Median:4	67	59	62	98	Cohort studies	OR/CR/PR/PFS/OS/CRS/ICANS
			n=82	n=149	Axi-cel Median:3						
Benoit 24	2023	R/R LBCL	Tisa-cel	Axi-cel	≥2	67	59	11pts in 2 Arms		Cohort studies	OR/CR/PR/CRS/ICANS
			n=10	n=15							
Locke 25	2021	R/R LBCL	SOC	Axi-cel	1	NA	NA	NA	NA	RCT	OR/CR/PR
			n=179	n=180							
Kamdar 26	2021	R/R LBCL	SOC	Liso-cel	1	NA	NA	NA	NA	RCT	OR/CR/PR
			n=92	n=90							
Bishop 27	2021	R/R NHL*	SOC	Tisa-cel	1	NA	NA	NA	NA	RCT	OR/CR/PR
			n=162	n=160							

Note: LBCL, large B-cell lymphoma; Liso-cel, lisocabtagene maraleucel;SOC, standard-of-care: SOC refers to the established treatments that medical professionals generally agree are appropriate and widely used for a particular type and stage of cancer; RCT, randomized controlled trial.* The major subtype in this study was diffuse large B-cell lymphoma-not otherwise specified, followed by high-grade B-cell lymphoma, primary mediastinal B-cell lymphoma, and follicular lymphoma grade 3B. bridging treatments (systemic treatment, radiotherapy, combined modality treatment)

**Table 2:** Results of the quality evaluation in six articles

Author	Year	Cohort selection	Comparability	Outcome	Total score
Bachy 19	2022	★★★★	★★	★★	8
Kuhnl 20	2022	★★★	★★	★★	7
Kwon 21	2022	★★★★	★★	★★	8
Bastos-Oreiro 22	2022	★★★	★★	★★	7
Riedell 23	2022	★★★	★★	★★	7
Benoit 24	2023	★★★★	★★	★★	8

**Table 3:** Other common adverse toxicities

Author	Year	Population	Treatment regimen		Neutropenia (any Grade/Grade ≥3)		Anemia (any Grade/Grade ≥3)		Thrombocytopenia (any Grade/Grade ≥3)	
			Arm A	Arm B	Arm A	Arm B	Arm A	Arm B	Arm A	Arm B
Bachy 19	2022	R/R DLBCL	Tisa-cel	Axi-cel	57/20	124/53	58/0	94/4	62/19	116/46
			n=209	n=209						
Kuhnl 20	2022	R/R LBCL	Tisa-cel	Axi-cel	NA/4*	NA/22*	NA	NA	NA/4*	NA/15*
			n=76	n=224						
Kwon 21	2023	R/R DLBCL	Tisa-cel	Axi-cel	116/22	110/31	NA	NA	115/46	104/49
			n=127	n=134						
Bastos-Oreiro 22	2022	R/R LBCL	Axi-cel	Tisa-cel	NA	NA	NA	NA	NA	NA
			n=101	n=91						
Riedell 23	2022	R/R LBCL	Tisa-cel	Axi-cel	NA	NA	NA	NA	NA	NA
			n=82	n=149						
Benoit 24	2023	R/R LBCL	Tisa-cel	Axi-cel	NA/1	NA/6	NA/0	NA/2	NA/1	NA/5
			n=10	n=15						
Locke 25	2021	R/R LBCL	SOC	Axi-cel	From 63rd ASH Abstracts:Grade ≥3treatment-emergent adverse events :155 (axi-cel) and 140 (SOC) pts					
			n=179	n=180						
Kamdar 26	2021	R/R LBCL	SOC	Liso-cel	49/NA	75/NA	58/NA	58/NA	62/NA	53/NA
			n=92	n=90						
Bishop 27	2021	R/R NHL	SOC	Tisa-cel	NA	NA	NA	NA	NA	NA
			n=162	n=160						

Note: *Cytopenia at 3 months
