# Supplementary material for: Comparison of the Efficacy and Safety of Axi-Cel and Tisa-Cel Based on Meta-Analysis
Source: J Cancer. 2024 Sep 9;15(17):5729–41. doi: 10.7150/jca.99427 (PMC11414620; doi:10.7150/jca.99427)
Supplement: Supplementary file 1 — Supplementary figure. [file jcav15p5729s1.pdf]

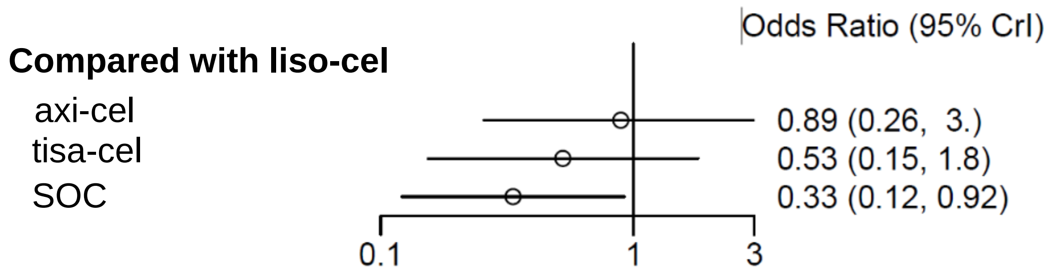

supplementary figure:Complete response rates (CRRs) of liso-cell ,tisa-cel ,axi-cel and SOC treatments
